# 3D localization from 2D X-ray projection

**DOI:** 10.1007/s11548-022-02709-w

**Published:** 2022-07-11

**Authors:** Dagmar Bertsche, Volker Rasche, Wolfgang Rottbauer, Ina Vernikouskaya

**Affiliations:** Department of Internal Medicine II, Ulm University Medical Center, Ulm, Germany

**Keywords:** Monoplane projection, 3D localization, Centerline model, Cerebral embolic protection

## Abstract

**Purpose:**

Most cardiology procedures are guided using X-ray (XR) fluoroscopy. However, the projective nature of the XR fluoroscopy does not allow for true depth perception as required for safe and efficient intervention guidance in structural heart diseases. For improving guidance, different methods have been proposed often being radiation-intensive, time-consuming, or expensive. We propose a simple 3D localization method based on a single monoplane XR projection using a co-registered centerline model.

**Methods:**

The method is based on 3D anatomic surface models and corresponding centerlines generated from preprocedural imaging. After initial co-registration, 2D working points identified in monoplane XR projections are localized in 3D by minimizing the angle between the projection lines of the centerline points and the working points. The accuracy and reliability of the located 3D positions were assessed in 3D using phantom data and in patient data projected to 2D obtained during placement of embolic protection system in interventional procedures.

**Results:**

With the proposed methods, 2D working points identified in monoplane XR could be successfully located in the 3D phantom and in the patient-specific 3D anatomy. Accuracy in the phantom (3D) resulted in 1.6 mm (± 0.8 mm) on average, and 2.7 mm (± 1.3 mm) on average in the patient data (2D).

**Conclusion:**

The use of co-registered centerline models allows reliable and accurate 3D localization of devices from a single monoplane XR projection during placement of the embolic protection system in TAVR. The extension to different vascular interventions and combination with automatic methods for device detection and registration might be promising.

## Introduction

Visualization of the exact position of the catheter or other device in the patient-specific anatomy has proven to improve navigation in catheter-based interventions as well as to enable documentation of target or working points for quality assurance. A key component is the 3D localization of the device. Electromagnetic (EM) tracking has been suggested [1–4], which requires dedicated, often expensive equipment. Alternative, 3D localization of the 3D position from biplane X-ray (XR) projections has been suggested and proven accurate [5–9]. However, this approach demands quasi-simultaneous projection from two directions, which may cause a substantial increase of radiation dose and limits its application to biplane XR systems. To enable 3D localization on monoplane systems, localization methods based on multiple subsequently acquired projections [10–13] or based on a continuously rotating C-arm [14] have been reported. Even though enabling 3D localization on monoplane systems, radiation exposure is still an issue and the non-simultaneous acquisition may cause inaccuracies due to organ or patient motion. The use of patient-specific vascular roadmaps e.g. acquired by 3D rotational angiography has been suggested and evaluated in phantom datasets to enable 3D guidewire reconstruction from a single monoplane XR projection [15–18]. In addition to phantom evaluation, qualitative patient data evaluation of monoplane-based 3D guidewire reconstruction based on surface backprojection has been described in two neuro-endovascular data sets [19], but lack further quantitative evaluation on patient data. Alternative to XR based localization, deep learning algorithms for instrument localisation in periprocedural 3D ultrasound have been reported [20, 21]. Deep learning approaches to derive 3D information from 2D XR images have so far focused on the reconstruction of bone [22] or vascular [23] structures without considering the localisation of devices.

In this contribution, we evaluate a 3D localization method based on a single monoplane projection. The approach is based on using preprocedurally derived patient-specific centerline anatomy models, which are registered to the XR geometry. The 3D accuracy of the approach was evaluated in a phantom with 3D ground truth information. Further in vivo data evaluation was performed in 16 patient datasets obtained during placement of the embolic protection system in transcatheter aortic valve replacement (TAVR) procedures. With the shown accuracy of this rather simple and straightforward approach its application to other, mainly transvascular, procedures appears feasible.

## Methods

For 3D device localization estimation, we propose the minimization of the angle between the projection lines of the centerline points and the point of the 2D device position in the respective XR image, which is defined the 3D location in the following. As such, a 3D model with respective centerline of the vascular target structure as well as an accurate registration between the 3D model and the XR system is required. The accuracy of the 3D localization was evaluated in a 3D printed phantom and based on 16 patient datasets in which cerebral embolic protection devices were implanted during TAVR. The required 3D models were derived from preprocedural CT data routinely acquired for intervention planning.

### General method

**Fig. 1:**
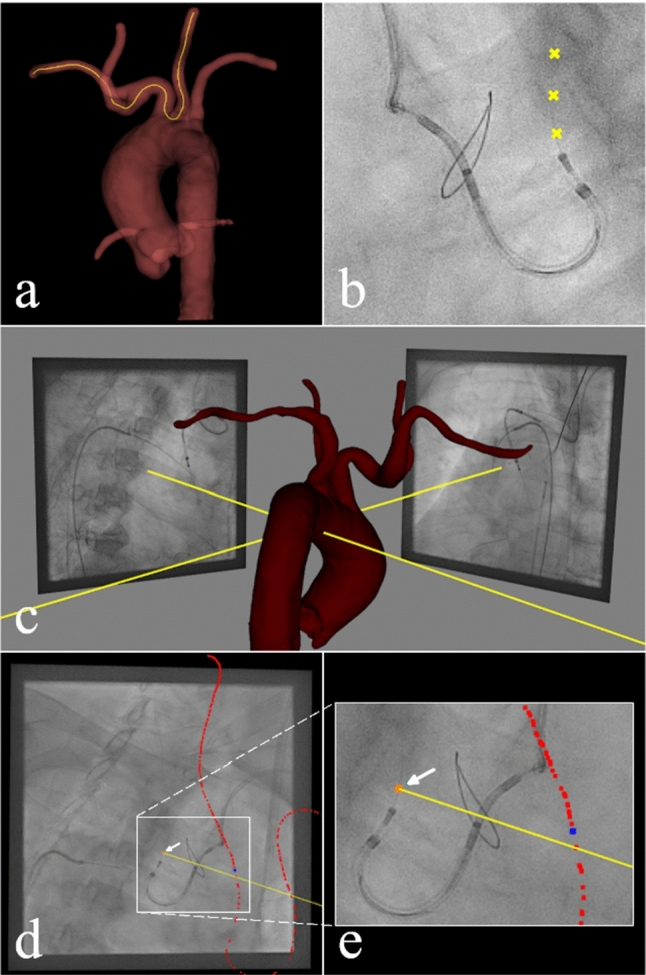
General method: (**a**) Extraction of centreline (orange line) of right subclavian artery and left common carotid artery from the aortic model generated from the preprocedural CT using 3DSlicer. (**b**) Example cut-out of placed markers (yellow) along the guidewire in the left common carotid artery in an x-ray projection. (**c**) Co-registration of preprocedural data and peri-procedural x-ray system based on two monoplane projections of the aortic arch with different angulations. (**d**) 3D localization example of one 2D marker (orange, arrow): determination the point (blue point) of the centreline (red points) whose projection line is most parallel to the projection line (yellow line) of the 2D marker. (**e**) Close-up of the white box in (**d**) for better visualization

Using 3DSlicer (www.slicer.org, [24]), surface meshes of the TAVR relevant vascular structures were generated from preprocedural computer tomography (CT) image volumes acquired for interventional planning as previously reported [25]. From these 3D surface meshes, Voronoi model-based centerlines [26] of the vascular structures were extracted as shown in Fig. 1a. The approach was evaluated retrospectively on monoplane XR projections (Allura Xper, Philips Medical Systems, Best, The Netherlands). Registration of the model with the XR coordinate system was performed using 3D-XGuide [27] based on successively acquired projections of the target anatomy (Fig. 1c). The projected position ${\varvec{p}}_{{2{\varvec{D}}}} \user2{ }$ of the 3D target positions ${\varvec{p}}_{{3{\varvec{D}}}}$ were identified manually in the XR projections of the common carotid artery (Fig. 1b). According to established camera models [28–30], the position of ${\varvec{p}}_{{2{\varvec{D}}}}$ on the detector in 3D XR system coordinates $p\prime_{2D}$ was calculated. Using the cross-product the point ${\varvec{c}}$ of the centerline ${\varvec{C}}$ whose ray to the position of the XR source ${\varvec{s}}$ is the most parallel with the projection line defined by $p\prime_{2D}$ and the position of ${\varvec{s}}$ in XR system coordinates was determined. Therefore, ${\varvec{c}}$ was defined as the localized 3D position ${\varvec{p}}_{{3{\varvec{D}}}}$ of ${\varvec{p}}_{{2{\varvec{D}}}}$ according to $p_{3D} : = \arg {\mathop {\min }\limits_{c \in C} \| \left( {c - s} \right)x\left( {p^{\prime}_{2D} - s} \right)} \|_{2}^{2}$. The methodology is exemplified in Fig. 1d and e.

A general limitation in projective data rises from ambiguities introduced by superimposed vascular structures, which may cause inconsistencies in the derived device path. To ensure consistent 3D locations, based on the initial 3D position approved by the operator, continuous movement of the device was ensured by restricting new 3D localization to centerline points in direct proximity to previous position.

### Phantom data

**Fig. 2:**
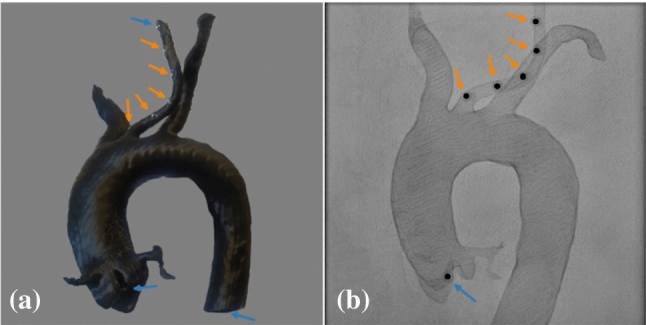
Phantom data: (**a**) printout of the aortic 3D model and (**b**) example projection of the phantom including markers for registration (blue arrows) and localization (orange arrows)

A surface model derived from a patient CT data set was 3D printed with GreenTec Pro (extrudr, FD3D GmbH, Lauterach, Austria) filament using the 3D printer RAISE3D Pro2 Plus and the associated software ideaMaker 4.0.1 (Raise3D, Irvine, California, USA). XR-opaque markers were attached at predefined locations within the left common carotid artery, the aortic root, and the descending aorta (Fig. 2a). XR projections of the 3D phantom were acquired with various angulations, mimicking typical projection geometries during a TAVR procedure. Affine 3D-3D registration of the model and the XR space was performed based on the known position of three fiducial markers located in the aortic root, the descending aorta, and the upper one of the markers located in the left common carotid artery and their reconstructed positions based on two monoplane projections. The remaining five markers in the left common carotid artery were selected manually in a further monoplane projection (Fig. 2b) and subsequently localized in 3D as described above. As reference ground truth the 3D marker location was further derived from two projections using epipolar geometry. Differences are presented as mean (m), standard deviation (std), median (mdn), and maximum (max) of the registration error, which is defined as the Euclidean distances in 3D (3D-ED) between the predefined fiducial marker positions after registration and the reconstructed fiducial positions, and the localization error defined as the 3D-ED between the single-monoplane-based 3D locations and the reference.

### Patient data

16 TAVR cases including cerebral embolic protection device implantation were randomly selected. Registration of the preprocedural aortic surface models with the XR system was performed manually based on two successively projections of the aortic arch with an angular distance of at least 30°. For each case, 20 points along the placed guidewire in the common carotid artery were manually identified in the projection documenting the implantation of the cerebral embolic protection device. The resulting 320 marked points were localized in 3D using the respective registered centerline. Since no 3D reference points were available, the accuracy was evaluated by the deviation of the localized 3D positions and previously marked 2D positions, both, projected to the plane in the iso-center parallel to the image plane. The mean (m), standard deviation (std), median (mdn), and maximum (max) of the resulting Euclidean distance in 2D (2D-ED) are reported.

## Real-time evaluation setup

Even though the main objective of this manuscript is to describe a simple approach for 3D device localization from monoplane XR data, its full potential e.g. for intervention navigation results from its combination with automatic device tracking. For demonstration, we combined the suggested approach with automatic device tracking by simple cross-correlation. Given the initial position of the cerebral embolic protection device marker in a patient 2D XR, the device marker was tracked automatically in the following XR frames and localized in 3D as described above.

## Results

**Fig. 3:**
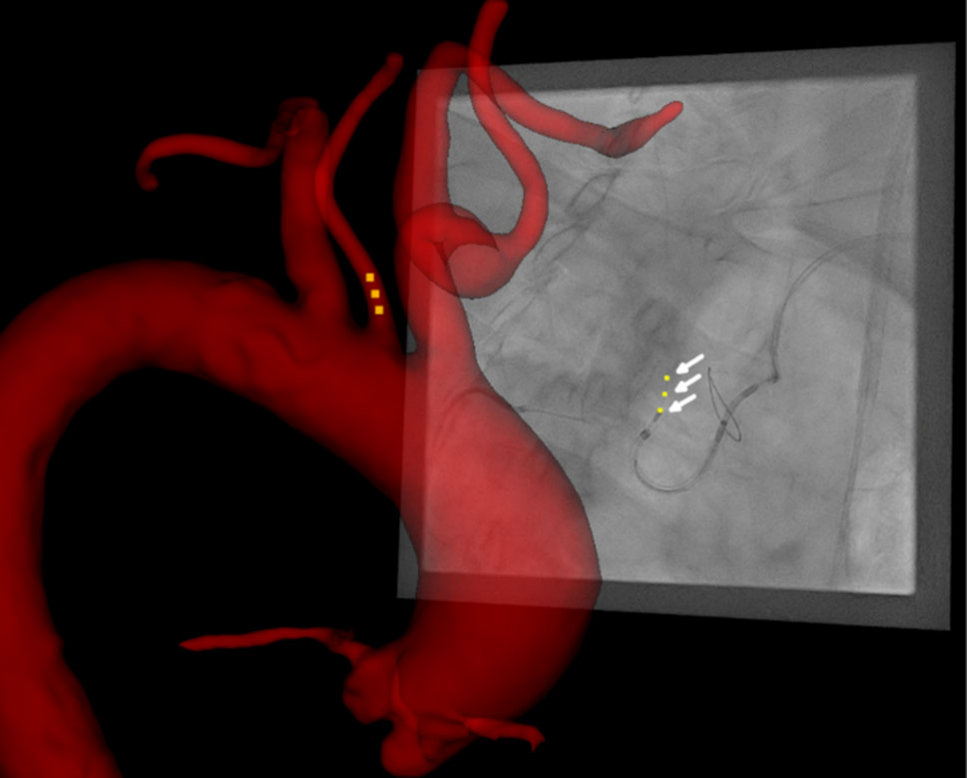
Result: Example of identified 2D positions in XR (yellow markers, white arrows) are localized and visualized in 3D (orange markers) in relation to patient specific anatomic model (red structure)

The 3D position of a device can be retrieved and superimposed to a registered vascular model from a single 2D projection (Fig. 3). In the phantom data, all 2D points defined in a single monoplane XR projection were localized in 3D in the correct vessel branch with a 3D-ED of m = 1.6 mm, std = 0.8 mm, mdn = 1.3 mm, max = 2.9 mm to the respective 3D reference positions. The registration error of the phantom dataset was a 3D-ED of m = 0.6 mm, std = 0.3 mm, mdn = 0.6 mm, max = 0.9 mm. Of the 320 points in the patient data, 97.5% were localized within the correct artery branch. In one patient data set 8 of the 20 points were determined within the wrong artery due to superimposed centerlines. Applying the continuity restriction, all points in this data set were also localized in the correct artery branch. The assessment of the patient data accuracy resulted in a 2D-ED of m = 2.7 mm, std = 1.3 mm, mdn = 2.5 mm, max = 8.1 mm. In the patient datasets, which included an average of 530 centerline points, each 3D localization took 0.6 ms on average, resulting in around 1.2 microseconds per centerline point. The automatic tracking of the device marker succeeded in the exemplified XR run. The 3D localization based on the 60 automatic tracked 2D positions resulted in an 2D-ED of m = 3.1 mm, std = 2.3 mm, mdn = 3.5 mm, max = 5.6 mm, with an average computation time of 23.5 ms on average per frame.

## Discussion

The evaluation of the phantom data demonstrated the feasibility of 3D localization from a single monoplane projection with high accuracy. Previous work [31] reported a required navigation accuracy of at least 5 mm for endovascular navigation, which indicates a sufficient accuracy of the proposed approach for 3D guidance and documentation of working points. However, it should be explicitly noted that the deviation in the patient data could only be assessed as a 2D projected distances due to the lack of ground truth data.

As the guidewire does not necessarily have to be in the center of the vessel, a maximal intrinsic possible error in the localization results to the vessel radius and may limit the proposed localization method to small diameter vessel interventions. The expected maximal inaccuracy for the common carotid artery is ~ 3.2 mm [32]. The mean errors in both the phantom and patient data resulted less than the maximal inaccuracy expected.

As a specific centerline point has to be identified, the precision and sampling distances of the centerline also affect the accuracy of the localization with a maximal expected deviation in the order of half the distance between two centreline points, which should be chosen smaller as the resolution in the XR projection.

Registration of the preprocedural model with the XR system is essential for the accuracy of the localization. The small registration error measured in the phantom data may have been caused by inaccuracies of the 3D-printing, the camera model obtained from the XR system or of the manual identification of the 2D points. Since the patient data lacked easily accessible landmarks for 3D-3D registration, manual registration was performed without possible quantitative accuracy assessment. The manual registration may be overcome by improved registration methods as reported previously [33, 34]. Refinement of the registration in course of an intervention may be necessary due to patient motion. Advanced motion compensation approaches [9, 35, 36] could make the monoplane 3D localization based on centerlines more robust and suitable for other vascular procedures, in which the target anatomy experiences cardiac and/or respiratory motion such as chronic total occlusions. Further small anatomic deformations between pre-interventional and interventional data have to be considered as a general limitation of using anatomic models for intervention guidance.

Unlike image fusion of e.g. of echocardiography and XR, where the echo probe can be used as registration landmark for automatic 2D-3D registration, CT-XR image fusion is normally based on manual 2D-3D registration. Thus, the proposed 3D localization could act as a real-time adjunct for 3D intervention guidance and 3D documentation of working points. However, the still required continuous manual identification of the 2D locations appears problematic, and needs automation. Therefore, the automatic tracking of an initial marker was exemplified, resulting in successful tracking of the device position in 2D and subsequent 3D localization in real-time. Even though the tracking was exemplified applying cross-correlation, the localization method is applicable in general and could be combined with other automatization methods as previously suggested [37–40].

Only the predetermined centerline tree is used to localize the 3D position using the proposed method and deviations from the tree are not reflected. Minimizing the angle between the projection lines does not consider the distance between XR source and detector by localizing the 3D position. Thus, for centerlines that are approximately parallel to the projection direction or superimposed centerlines, the 3D localization may fail. As orientation in XR suffers from centerlines parallel to the projection direction or superimposed centerlines of the relevant artery branches in the working area, projection angulations are chosen to avoid such centerline constellations in the relevant area. However, patient-specific anatomy or procedure-dependent pathways may cause centerlines of previously relevant areas to be superimposed in projection with later relevant areas. To avoid limitations caused by superimposed centerlines, regularization prohibiting noncontinuous motion between subsequent XR projections might be required as suggested. Further patient-specific pre-procedural projection planning could prevent centerlines parallel to the projection direction or superimposed centerlines and enable a clear anatomical view.

## Conclusion

3D localization based on a single monoplane projection augmented by an anatomic centerline model has been successfully performed with sufficient accuracy for 3D navigation, exemplified for placing the embolic protection device during TAVR. Due to intrinsic limitations in the accuracy mainly due to uncertainty of the location of the device in the vascular structure, the application to different vascular interventions has to be further evaluated. In combination with automated device identification, an almost automatic real-rime 3D guidance appears feasible.
